# The Role of Forkhead Box O in Pathogenesis and Therapy of Diabetes Mellitus

**DOI:** 10.3390/ijms231911611

**Published:** 2022-10-01

**Authors:** Malgorzata Marchelek-Mysliwiec, Magdalena Nalewajska, Agnieszka Turoń-Skrzypińska, Katarzyna Kotrych, Violetta Dziedziejko, Tadeusz Sulikowski, Andrzej Pawlik

**Affiliations:** 1 Department of Nephrology, Transplantology and Internal Medicine, Pomeranian Medical University, 70-204 Szczecin, Poland; 2 Department of Medical Rehabilitation and Clinical Rehabilitation, Pomeranian Medical University, 70-204 Szczecin, Poland; 3 Department of Radiology, West Pomeranian Center of Oncology, Pomeranian Medical University, 70-204 Szczecin, Poland; 4 Department of Biochemistry and Medical Chemistry, Pomeranian Medical University, 70-204 Szczecin, Poland; 5 Department of General, Minimally Invasive, and Gastroenterological Surgery, Pomeranian Medical University, 70-204 Szczecin, Poland; 6 Department of Physiology, Pomeranian Medical University, 70-204 Szczecin, Poland

**Keywords:** FOXO, diabetes, therapy

## Abstract

Type 2 diabetes is a disease that causes numerous complications disrupting the functioning of the entire body. Therefore, new treatments for the disease are being sought. Studies in recent years have shown that forkhead box O (FOXO) proteins may be a promising target for diabetes therapy. FOXO proteins are transcription factors involved in numerous physiological processes and in various pathological conditions, including cardiovascular diseases and diabetes. Their roles include regulating the cell cycle, DNA repair, influencing apoptosis, glucose metabolism, autophagy processes and ageing. FOXO1 is an important regulator of pancreatic beta-cell function affecting pancreatic beta cells under conditions of insulin resistance. FOXO1 also protects beta cells from damage resulting from oxidative stress associated with glucose and lipid overload. FOXO has been shown to affect a number of processes involved in the development of diabetes and its complications. FOXO regulates pancreatic β-cell function during metabolic stress and also plays an important role in regulating wound healing. Therefore, the pharmacological regulation of FOXO proteins is a promising approach to developing treatments for many diseases, including diabetes mellitus. In this review, we describe the role of FOXO proteins in the pathogenesis of diabetes and the role of the modulation of FOXO function in the therapy of this disease.

## 1. Introduction

Forkhead box class O proteins (FOXO) belong to a family of transcription proteins that are involved in many cellular processes and are, therefore, important links in the regulation of cell homeostasis. The FOXO family comprises approximately 100 proteins classified alphabetically from A to R. In mammals, the O group includes the following transcription factors: FOXO1, FOXO3, FOXO4 and FOXO6. Their roles include regulating the cell cycle, DNA repair, influencing apoptosis, glucose metabolism, autophagy processes and ageing [1]. FOXOs respond to a wide range of external stimuli such as growth factors, oxidative stress, genotoxic stress and nutrient deprivation. These interactions are crucial in coordinating the body’s response to environmental changes and maintaining cellular homeostasis in a broad sense.

Such multidirectional activity is possible due to the post-translational modifications that FOXO undergoes: phosphorylation, acetylation, ubiquitination, methylation and glycosylation.

The importance of FOXO proteins has been proven in a number of pathophysiological processes such as diabetes, cardiovascular diseases, cancer and neurodegenerative diseases; therefore, they are being investigated as therapeutic targets for the above-mentioned disorders.

## 2. Structure of FOXO

**Figure 1:**
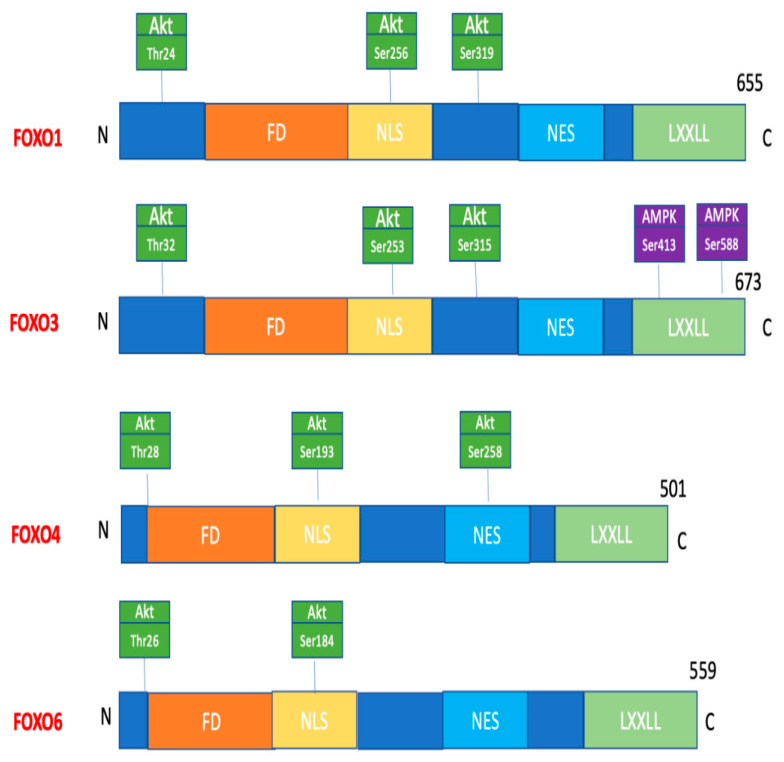
Description of mammalian FOXO proteins expressed in skeletal muscle. The following are indicated: locations of the forkhead domain (FD), nuclear localisation sequence (NLS), nuclear export sequence (NES) and helical motif (LXXLL), Akt phosphorylation sites (green squares) and AMPK phosphorylation sites (purple squares) [4].

FOXO proteins have a conserved part that binds to cellular DNA and four motifs: forkhead DBD, nuclear localisation signal (NLS), nuclear export signal NES (and) transactivation domains (TAD) (Figure 1).

The sequences responsible for nuclear localisation and nuclear export are located on the C-terminal DNA-binding domain [2].

The post-translational changes that FOXO undergoes enable it to reach a precise location in the cell nucleus and initiate transcriptional activity. On the other hand, the presence of 14-3-3 proteins allows the active export of FOXO from the nucleus and the inhibition of its reassembly with DNA [3].

## 3. Post-Translational Changes—A Brief Overview

### Phosphorylation

FOXO phosphorylation is an important mechanism for regulating FOXO transport to and from the cell nucleus. Several kinases are involved in the phosphorylation of FOXO proteins: AKT (serine/threonine kinase), SGKs (serum/glucocorticoid regulated kinase), PERK (PKR-like ER kinase), MST1 (mammalian sterile 20-like kinase), JNK, (Jun-N-terminal kinase), CDK1/2 (cyclin-dependent kinase), ERK/p38 (extracellular signal-regulated kinase), AMPK (adenosine monophosphate-activated protein kinase) and IK (IK kinase). Each of them modifies FOXO at a specific site in a characteristic manner (Table 1). Depending on the kinase and the site of phosphorylation, FOXO either undergoes translocation to the nucleus or is retained in the cytoplasm [5].

## 4. Acetylation and Deacetylation of FOXO

The acetylation of FOXO, similar to phosphorylation, leads to changes in the DNA strand-binding properties of these proteins. In general, acetylation impairs the transcriptional activity of FOXO, whereas under stress conditions, it stabilises FOXO and prevents degradation by ubiquitination [6].

The opposite process, the deacetylation of FOXO, occurs through sirtuins and HDACs, among others, in response to oxidative stress. Sirtuins have dual effects; they can increase or decrease the transcriptional capacity of FOXO, either through retention in the cytoplasm or translocation into the cell nucleus [7].

## 5. Ubiquitination

Ubiquitination is another process of enzymatic post-translational modification of proteins. It involves the attachment of molecules of a small protein, ubiquitin, which occurs in the cytoplasm and the nucleus of cells. The result of ubiquitination depends on the amount of attached ubiquitin [8]. The attachment of ubiquitin monomers, monoubiquitination, results in an increase in FOXO activity. Polyubiquitination causes the protein labelled in this way to enter the proteasome before it is degraded. FOXO degradation involving ubiquitination depends on the ubiquitin–proteasome pathway [9].

## 6. Methylation

The methylation of FOXO at positions Arg248 and Arg250 inhibits the Akt-induced transformation of FOXO. As a result, FOXO remains in the cell nucleus [10].

## 7. Glycosylation

Glycosylation involves the attachment of carbohydrates to organic compounds, including proteins. The attachment of a sugar moiety to a protein occurs through an N- or O-glycosidic bond. FOXO 1 is the substrate for the O-glycosylation process. This process does not affect the transport of FOXO from the nucleus to the cytoplasm [11].

## 8. Role of FOXO Proteins in Diabetes and Its Complications

**Figure 2:**
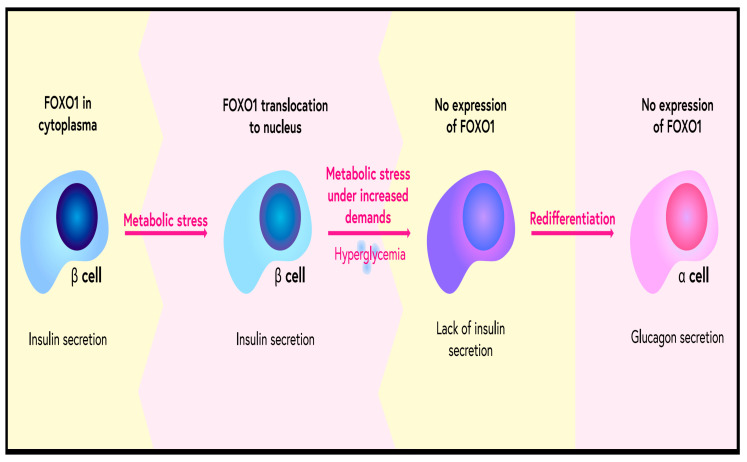
Role of FOXO1 in metabolic stress.

Type 2 diabetes affects a growing number of people; when poorly controlled, it causes numerous complications involving the functioning of the whole body. Cardiovascular, ocular and renal complications are well known. The cause of diabetes is both a dysfunction in insulin secretion by the beta cells of the pancreas and a disturbance in insulin action in tissues leading to insulin resistance. FOXO proteins can affect both insulin synthesis in pancreatic beta cells and can also affect insulin resistance. FOXO1 is an important regulator of pancreatic beta-cell function. FOXO1 influences pancreatic beta cells under conditions of insulin resistance and during their differentiation in the developing foetal pancreas. FOXO1 also protects beta cells from damage resulting from oxidative stress associated with glucose and lipid overload [12] (Figure 2). Blocking FOXO1 function has been shown to result in impaired pancreatic beta-cell function [13]. In healthy individuals, FOXO transcription factors remain inactive in beta cells and become activated in response to hyperglycaemia [14]. In patients with type 2 diabetes, loss of FOXO in beta cells correlates with a loss of insulin secretion capacity [15]. In the early stages of diabetes, FOXO transcription factors remain inactive. Animal studies have shown that this is due to an attempt to maintain a balance between acetyl-coenzyme A (Acetyl-CoA) synthesis from glucose and synthesis from lipids. Acetyl-CoA is essential for mitochondrial oxidation [16]. In the early phase of diabetes, glucose oxidation is impaired compared to beta cells of healthy individuals, while lipid oxidation is significantly increased. Excessive lipid oxidation leads to the formation of toxic products, mainly peroxides, impaired ATP synthesis and impaired insulin secretion [17]. Chronic oxidative stress in diabetes is likely induced by excessive cellular glucose concentrations that exceed the glycolytic capacity of beta cells. Under these conditions, glucose is enolysed, resulting in the synthesis of superoxide anions and induction of apoptosis of these cells [18]. Oxidative stress enhances FOXO1 activation, which protects beta cells from acute metabolic disturbances and damage caused by short-term oxidative stress but does not protect against chronic changes associated with long-term hyperglycaemia [19]. FOXO1 also enhances the expression of the enzymes catalase and glutathione peroxidase in beta cells, leading to better function of these cells during oxidative stress and increased survival. Previous studies have shown an important function for FOXO in protecting pancreatic beta cells from oxidative stress, which are particularly susceptible to the effects of oxidative stress due to their low expression of antioxidant enzymes [20]. FOXO1 has been shown to protect β-cells from oxidative stress by inhibiting the expression of the thioredoxin interacting protein (TXNIP), a cytosolic factor whose deregulation is associated with β-cell apoptosis [21]. FOXO1 also enhances the expression of the DNA repair enzyme GADD45α, protecting β-cells from nitric-oxide-induced DNA damage [22].

FOXO1 also plays an important role in regulating muscle energy homeostasis. FOXO1 is an important regulator of glucose metabolism in skeletal muscle, as well as in adipose tissue, liver, pancreas and bone [23]. FOXO1 has been shown to affect muscle energy homeostasis by reducing carbohydrate catabolism during caloric deficiency [24]. FOXO1 regulates the expression of pyruvate dehydrogenase kinase 4 (PDK4), involved in maintaining blood glucose levels [25]. High levels of pyruvate dehydrogenase kinase result in a decrease in pyruvate dehydrogenase (PDH) activity, which is responsible for utilising carbohydrates as an energy substrate. Thus, the FOXO1-induced increase in pyruvate dehydrogenase kinase 4 expression results in the preservation of glucose and gluconeogenesis substrates such as lactate, pyruvate and alanine [26]. FOXO1 also affects the transport of fatty acids into muscle cells. FOXO1 is an important regulator of lipid oxidation in muscle. FOXO1 has been shown to stimulate lipoprotein lipase (LPL), an enzyme that hydrolyses plasma triglycerides into fatty acids and glycerol for uptake by muscle cells [27]. In addition, FOXO1 upregulates the expression of adiponectin receptors (AdipR), enhancing fatty acid oxidation in muscle and increasing adiponectin sensitivity [28]. Together, these data show that FOXO1 causes the use of lipids instead of glucose as an energy substrate under stress conditions. FOXO1 appears to be an important regulator of muscle energy homeostasis by regulating glucose and lipid metabolism.

FOXO1 plays a key role in the regulation of hepatic glucose and lipid synthesis. FOXO1 controls the metabolic switch between glucose and lipid oxidation depending on food availability. In hyperinsulinemic mice, FOXO1 inhibition decreased glycogenolysis and gluconeogenesis, leading to increased tissue sensitivity to insulin [29]. Under conditions of insulin resistance, FOXO1 remains active and continues to affect microsomal triglyceride transfer protein (MTTP) and apolipoprotein C-III (APO C-III), which is one of the proteins responsible for hypertriglyceridemia [30].

**Figure 3:**
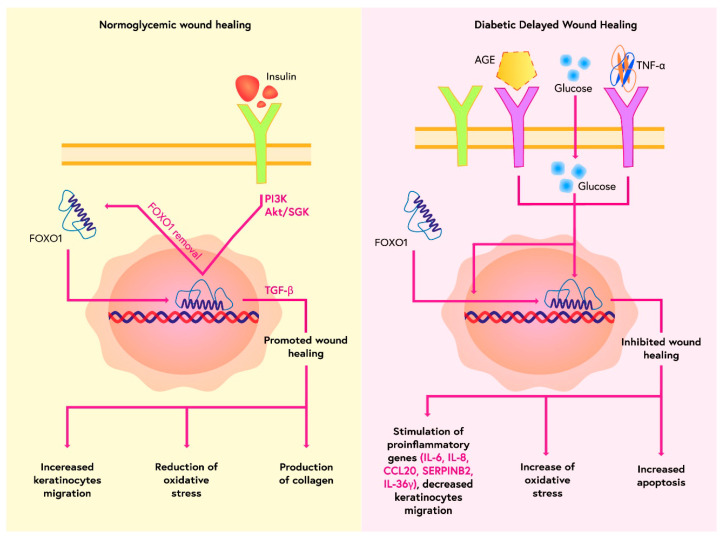
Role of FOXO1 in wound healing.

A significant problem for patients with diabetes is impaired wound healing (Figure 3). FOXO1 has been shown to play an important role in cellular processes related to wound healing [31]. FOXO1 is involved in the regulation of keratinocyte function [32]. FOXO1 deletion has been shown to delay wound healing as a result of a reduction in the number of migrating keratinocytes in both the wounded epithelium and hair follicles [33]. It was detected that the main factor regulated by FOXO1 is the expression of TGF-β1, which is essential for normal wound healing. Blocking FOXO1 has been shown to reduce TGF-β1 expression in keratinocytes [34]. On the other hand, TGF-β1 stimulates keratinocyte migration and enhances the wound healing process. FOXO1 has also been shown to promote other pathways involved in wound healing by stimulating integrins-α3 and -β6 and matrix metalloproteinases-3 and -9 [34]. Thus, it appears that treatment with FOXO1 agonists may represent a potential therapeutic target for wound healing by mobilising keratinocytes for rapid wound epithelialisation and protection against oxidative stress.

FOXO1 is translocated into the β-cell nucleus during metabolic stress. However, severe metabolic stress and increased demands could lead to a lack of FOXO1 and insulin expression by β-cells. In such conditions, β-cells undergo a redifferentiation process. Some start to remind α-cells as they start to express glucagon.

FOXO1 has an essential role in the regulation of wound healing. In normoglycemic conditions, FOXO1 increases the transcription and expression of transforming growth factor β1 (TGF-β). TGF-β activates keratinocytes, promoting wound healing. The process is also regulated by insulin through the Akt and PI3K pathways, which remove FOXO1 from the nucleus, preventing its hyperactivation. In diabetic conditions, there is no regulation by insulin. Glucose, advanced glycation end products (AGEs) and tumour necrosis factor α (TNF-α) promote FOXO1 movement into the nucleus. FOXO1 increases TGF-β1 expression in keratinocytes. TGF-β1 stimulates keratinocyte migration and enhances the wound healing process. Moreover, FOXO1 is also modulated by those factors, so it elevates the expression of chemokine ligand 20 (CCL20), serpin peptidase inhibitor (SERPINB2) and interleukin 36ϒ (IL-36ϒ). Those products influence keratinocytes and disturb the process of re-epithelialisation and wound healing.

## 9. FOXO as a Therapeutic Target in Type 2 Diabetes and its Complications

### 1,25-Dihydroxyvitamin D

1,25-dihydroxyvitamin D (1,25 D) is the most biologically active form of vitamin D. As a nuclear hormone, 1,25 D participates in the regulation of gene transcription by binding to the vitamin D receptor (VDR). Prospective studies have clearly shown that 1,25 D reduces the risk of developing type 2 diabetes in predisposed individuals [35,36,37]. Furthermore, 1,25 D supplementation has been shown to reduce insulin resistance (IR) in individuals with prediabetes and IR [38,39,40] and improve insulin secretion, insulin sensitivity and HbA1c [41]. New studies have shown that 1,25 D can improve carbohydrate metabolism and reduce the risk of developing diabetic complications by decreasing the expression and nuclear exclusion of the FOXO1 protein. A study by Chen et al. demonstrated that 1,25 D-dependent VDR signalling inhibits FOXO1 expression, its nuclear translocation and activity in skeletal muscle cells in mice [42]. In the same study, VDR receptor blockade led to the loss of FOXO1 suppression, thus indicating that the suppressive effects of vitamin D are strictly dependent on the VDR receptor. In another study, 1,25 D was shown to benefit carbohydrate metabolism and bone turnover in mice by inhibiting FOXO1 expression through a PI3K/Akt-dependent pathway [43]. Through phosphorylation and the nuclear exclusion of the FOXO1 protein, 1,25 D led to increased osteoblast differentiation and increased total osteocalcin, as well as noncarboxylated osteocalcin. The study authors postulate that by this mechanism, vitamin D may at least partially reverse the negative effects of high glucose concentrations on bone metabolism. In a newly published study, Guo et al. demonstrated a protective role of 1,25 D on the diabetic heart in a rat model [44]. The activation of 1,25 D/VDR led to the blockade of FOXO1 nuclear translocation, decreased total and nuclear FOXO1 expression and the reduced severity of autophagy and apoptosis in the heart. In the same study, 1,25 D treatment had beneficial effects on carbohydrate metabolism, improving oral glucose tolerance test results, reducing fasting glucose levels and creatine kinase isoform CK-MB levels.

## 10. N1-Methylnicotinamide

N1-Methylnicotinamide (MNAM) is a product of nicotinamide methylation by the enzyme nicotinamide N-methyltransferase. Several studies to date have demonstrated the beneficial role of MNAM in the treatment of obesity and IR by modifying blood glucose and insulin levels [45,46]. Zhang et al. demonstrated that the administration of MNAM to obese diabetic mice is responsible for the regulation of hepatic gluconeogenesis, reducing fasting glucose and insulin levels [47]. MNAM treatment was associated with an increase in both the concentration and activity of the silent information regulator T1 (SITR-1) protein through its ubiquitination. SIRT1 reduced the transcriptional capacity of FOXO, thereby leading to reduced glucose production, reduced IR, reduced lipid accumulation and improved liver morphology, as shown in the cited study.

## 11. Fucoxanthin

Fucoxanthin (Fx) is an organic compound from the xanthophyll group that is a natural brown pigment. Among the numerous uses of Fx, its beneficial effects of normalising glycaemia and insulinaemia are mentioned [48]. Yang et al. recently evaluated the effects of Fx on oxidative stress and fibrotic processes in diabetic kidney disease and the role of FOXO3, Sirt1 and Akt kinase [49]. It has been shown that Sirt1 exerts anti-inflammatory effects and reduces the hyperglycaemia-induced fibrosis of glomerular mesangial cells by promoting the transcriptional activity of the FOXO3 protein [50,51]. FOXO3 is responsible for regulating the expression of genes responsible for protection against free radicals and is an important factor in the regulation of insulin secretion [52]. In the above study, the administration of Fx reversed the hyperglycaemia-induced deposition of the extracellular matrix in the kidney and reduced free radical generation, thereby inhibiting the progression of diabetic kidney disease. Fx also caused the reversal of Akt kinase activation and Sirt1 inhibition observed in the model studied, resulting in increased FOXO3 expression. The authors suggest that Fx may prove to be an effective treatment for diabetic kidney disease.

## 12. miR-233

MicroRNAs (miRNAs) are 20-nucleotide, single-stranded, noncoding molecules involved in the post-transcriptional regulation of the expression of numerous genes. Recently, a beneficial role of microRNAs in carbohydrate metabolism and pancreatic beta-cell development has been postulated [53,54]. A study by Li et al. [55] evaluated the role of microRNA-223 in maintaining pancreatic cell function. In this study, miR-223 was shown to positively affect pancreatic cell function and quantity through the regulation of FOXO1 and the developmental determinant factor Y-nucleus ((sex-determining region Y-box (Sox6)). The authors showed that miR-223 depletion leads to IR, the dysfunction of pancreatic cells and reduced cell proliferation. One of the mechanisms responsible is the decreased phosphorylation of Akt, leading to the decreased phosphorylation of FOXO1 and the increased accumulation of FOXO1 in the cell nucleus, which, in turn, resulted in the suppression of pancreatic and duodenal homeobox 1 (Pdx1) and glucose transporter 2 (Glut2) promoter and increased p27 protein expression. In turn, the overexpression of miR-223 in pancreatic cells improved the proliferation and function of these cells. The authors conclude that the inhibition of FOXO1 by the overexpression of miR-223 may lead to the improved function of pancreatic cells and contribute to the development of a new treatment for diabetes.

## 13. Entacapone

Entacapone is a medication used in Parkinson’s disease. It is a selective and reversible inhibitor of COMT (catechol-O-methyltransferase), an enzyme involved in the metabolism of levodopa. Its action is also based on the selective inhibition of FTO (fat mass and obesity protein). FTO is a recognised genetic risk factor for the development of obesity, and the FTO protein it encodes belongs to a group of mRNA-demethylating enzymes at the m6A (N6-adenosine-modified) and m6Am (N6,2’-O-dimethyladenosine-modified) sites. In a recently published study, Peng et al. [56] demonstrated that by regulating FOXO1 expression through the demethylation of FOXO1 mRNA at the m6A site, FTO is responsible for the regulation of hepatic gluconeogenesis. FTO deficiency induced by entacapone treatment resulted in decreased FOXO1 expression and, clinically, decreased body fat, increased energy expenditure and decreased fasting blood glucose levels.

## 14. Diazoxide

Diazoxide (DZX) is a benzothiadiazine derivative that acts by, among other things, activating mitochondrial ATP-dependent potassium channels (mitoKATP) by increasing cell membrane permeability to potassium ions. The opening of mitoKATP channels regulates the AKT–FOXO1 signalling pathway and leads to the formation of their phosphorylated forms: pAKT and pFOXO1. The result is decreased insulin secretion by pancreatic cells. As an activator of mitoKATP, DZX has been recognised as a cardioprotective factor. It affects the reduction in oxidative stress and apoptosis and the repair of ischaemia-reperfusion injury [57]. The expression of phosphorylated forms of AKT and FOXO1 has been shown to be decreased in diabetic cardiomyopathy [58]. A study by Duan et al. analysed the effect of DZX treatment on cardiomyocyte function in diabetic mice. They showed that the opening of mitoKATP channels and the phosphorylation of AKT-FOXO1 resulted in decreased plasma B-type natriuretic peptide (BNP) levels and improved left ventricular ejection fraction [59].

## 15. AS1842856

In 2010, researchers identified 5-amino-7-(cyclohexyl-amino)-1-ethyl-6-fluoro-4-oxo-1,4-dihydroquinoline-3-carboxylic acid (AS1842856) as a selective FOXO1 inhibitor [60]. In this study, the oral administration of AS1842856 was associated with a reduction in fasting plasma glucose levels and improved glucose tolerance in diabetic db/db mice [60]. The mechanism of action of AS1842856 is based on binding to the dephosphorylated, or active, form of FOXO1. Thus, inhibition of the transcriptional capacity of FOXO1 leads to reduced hepatic gluconeogenesis through the inhibition of mRNA glucose-6 phosphatase (glucose-6 phosphatase) and PEPCK (phosphoenolpyruvate carboxykinase) in vivo and in vitro. In a recently published study, we evaluated the effect of FOXO1 inhibition by AS1842856 on improving endothelial cell (EC) function and neovascularisation in a diabetic model. FOXO1 blockade was shown to be associated with improved blood flow and increased capillary density, resulting in an improvement in wound healing. Furthermore, the administration of AS1842856 resulted in decreased reactive oxygen species (ROS) free radical production and the inhibition of apoptosis induced by high glucose concentrations in ECs. The beneficial effect of FOXO1 inhibition could be achieved by modulation of the ROCK1-Drp1(Rho-associated coiled-coil containing protein kinase 1-dynamin-related protein-1) pathway [61]. Another study evaluated the effect of FOXO1 on stem cell differentiation towards insulin-producing cells. Successful in vitro differentiation of human embryonic stem cells (hESCs) into insulin-producing cells (IPCs) raises hope for the future use of this method in the treatment of diabetes. FOXO1 is believed to be one of the factors that negatively regulates the differentiation of hESCs into IPCs. Yu et al. showed that AS1842856 treatment induced the phosphorylation of FOXO1 and its export from the cell nucleus to the cytoplasm, thus inhibiting the FOXO1-dependent activation of genes involved in pancreatic islet cell differentiation. Simultaneously, AS1842856 had a beneficial effect on insulin secretion by IPCs. A similar effect was obtained by lentiviral silencing of FOXO1. The authors conclude that FOXO1 inhibition and knockout have beneficial effects on IPC maturation and glucose-dependent insulin secretion [62].

## 16. Atorvastatin

Atorvastatin is a representative of the group of statins, or HMG-CoA 3-hydroxy-3-methyl-glutaryl-coenzyme A) reductase inhibitors, used in the treatment of atherosclerosis. In addition to their hypolipemic effect, statins exhibit pleiotropic effects, including stabilising EC cell function. Their protective effect on EC homeostasis was assessed in a study by Park et al. The researchers demonstrated that high glucose levels inactivate Akt and Skp2 (S-phase kinase-associated protein 2) in EC cells. This is a dependent proteosomal degradation pathway of FOXO1 and ICAM-1 (intercellular adhesion molecule 1). The results of the inhibition of this pathway are the development of inflammation and endothelial destruction, which leads to the clinical manifestations of vascular complications of diabetes. The study also showed that atorvastatin treatment increased the binding of FOXO1 and ICAM-1 by Skp2, causing an increase in the ubiquitination and degradation of FOXO1 and ICAM-1 [63].

The beneficial effect of atorvastatin was also demonstrated in another study. In a high glucose environment, the activation of FOXO and inhibition of KLF2 (Kruppel-like factor 2) have been reported. KLF-2 is a factor found in ECs that is responsible for vasodilation and has anti-inflammatory, antithrombotic and antiproliferative effects. In the presence of atorvastatin, the FOXO protein was phosphorylated and shuttled into the cytoplasm; on the other hand, increased KLF 2 levels were also observed [64,65].

The authors suggest that the inactivation of FOXO1 by atorvastatin was due to an increase in its phosphorylation and the blocking of translocation into the cell nucleus.

## 17. Resveratrol

Resveratrol is an organic chemical compound that is a polyphenolic derivative of stilbene. Biological activity is demonstrated by the trans isomer. Resveratrol is primarily found in grapes, mainly in the skin, but also in other fruits such as blackcurrant and mulberry. Resveratrol has been shown to have the following properties: anti-inflammatory, reducing oxidative stress, inhibiting fibrosis and modulating cellular ageing processes, angiogenesis and platelet aggregation [66,67]. The mechanism of beneficial action involves activation of the SIRT1 gene.

A study by Wang et al. showed that resveratrol increased SIRT1 deacetylase activity, thereby reducing the expression of the acetylated form of FOXO3a and inhibiting hyperglycaemia-induced oxidative stress. In contrast, the silencing of SIRT1 resulted in the overexpression of the acetylated form of FOXO3, which potentiated hyperglycaemia-induced oxidative stress induced in renal tubules [68].

In contrast, in another study, Li et al. demonstrated that resveratrol also stimulates the fusion of FOXO1 with SIRT1. This resulted in a decrease in the acetylated form of FOXO1 and the consequently beneficial effects on cardiac remodelling, the inhibition of renal tubular damage and glomerular sclerosis in rats with chronic kidney disease [69].

## 18. Liraglutide

Liraglutide is a drug used to treat diabetes; this is an analogue of human glucagon-like peptide-1 (GLP-1). The drug improves glycaemic control by reducing fasting and postprandial blood glucose levels. A study by Chen et al. examined the effects of liraglutide on renal function in diabetic rats. Based on this study, it was not possible to demonstrate a direct mechanism for the effect of liraglutide on FOXO, but the conclusions that were obtained indicate that it may have a protective effect on renal function using FOXO1-mediated upregulation of renal MnSOD (manganese superoxide dismutase) expression in early DKD (diabetes kidney disease) [70].

## 19. Metformin

Metformin is a drug that has been used to treat diabetes for a very long time. In addition to its antihyperglycemic effect, it has many other proven beneficial effects, including its effect on lowering lipids and body weight and reducing the risk of cardiovascular death independent of glycaemic control. The mechanism of action of metformin involves effects on adenosine monophosphate-activated protein kinase, resulting in the inhibition of gluconeogenesis [71]. Experimental studies have shown that metformin effectively mitigated carbohydrate and lipid disturbances, reduced oxidative stress, improved renal function, enhanced autophagy and slowed the proliferation of abnormal cells under hyperglycaemic conditions through the AMPK/SIRT1–FOXO1 pathway [12].

## 20. Clinical Significance and Future Perspectives

Diabetes is a disease that leads to numerous complications, so proper therapy is very important. Studies to date suggest that FOXO may provide an opportunity for effective prevention and treatment of diabetes mellitus and its complications. Recent studies also indicate great potential for the clinical use of FOXO proteins in regenerative medicine. FOXO1 inactivation in intestinal endocrine cells has been shown to induce the formation of enteroendocrine progenitor cells containing neurogenin-3 and the emergence of functional insulin-producing cells that express all markers of mature pancreatic beta cells, including C-peptide, and release insulin in response to glucose [72]. Thus, it seems that blocking FOXO1 in the gut may be a promising strategy for the treatment of diabetes. FOXO proteins are essential for maintaining the normal differentiation process of pancreatic beta cells. The development of diabetes is associated with impaired differentiation of pancreatic beta cells. FOXO stabilises insulin-producing pancreatic beta cells and prevents their loss of function. Therefore, modulating FOXO proteins may be a promising method of preventing the development of diabetes [13]. However, the clinical application of FOXO proteins requires a thorough understanding of the role of FOXO in the many processes and pathways involved in the development of diabetes. Based on the studies performed so far, FOXO proteins can be described as a group of proteins with very versatile capabilities. Post-translational modifications are crucial, primarily for maintaining cellular homeostasis. Importantly, depending on the state of the body (e.g., oxidative stress or lack thereof), the same modification produces different effects at any given time. It is also not insignificant that FOXO proteins are associated with carbohydrate metabolism, among other things. Diabetes is a disease that is being diagnosed with increasing frequency and in younger and younger individuals. FOXO proteins offer hope for therapies, not only for glucose regulation itself but for a positive effect on delaying the onset of diabetes complications. Research should be conducted in this direction. High hopes are being pinned on the mechanism of the increased phosphorylation of FOXO by Akt and the consequent overexpression of miR-223 [73]. The use of FOXO proteins in clinical practice must be preceded by a further understanding of the mechanisms leading to the development of complications in diabetes and the role FOXO proteins play in them. In addition, it is very important to know through which factors FOXO proteins affect cellular metabolism. Several studies suggest that the inactivation of FOXO proteins may promote cytoprotection, reduce insulin resistance and increase pancreatic β-cell survival [13]. In contrast, other experimental studies suggest that increased expression of FOXO protein is beneficial for insulin signalling and maintenance of energy reserves [74]. It appears that epigenetic changes, as well as post-translational modifications of the FOXO protein under conditions of impaired cellular metabolism, may influence the directions of FOXO action in diabetes. Only a thorough understanding of the action of FOXO proteins in various states of cellular metabolic disorders can enable their use in the treatment of metabolic disturbances in diabetes.

## Figures and Tables

**Table 1:** Short characteristics of FOXO proteins.

Kinase/FOXO/Site of Phosphorylation	Physiological Effect of Phosphorylation
AKT FOXO1: Thr^24^, Ser^256^, Ser^319^ FOXO3: Thr^32^, Ser^253^, Ser^315^ FOXO4: Thr^28^, Ser^193^, Ser^256^ FOXO6: Thr^26^, Ser^184^	FOXO retention in the cytoplasm
SGKs/Ser^315^	FOXO retention in the cytoplasm
PERK/ Ser^298^ Ser^301^ Ser^303^	FOXO retention in the cell nucleus, increase in transcriptional activity
MST1/Ser^212^	Phosphorylation of FOXO3 translocation into the cell nucleus
AMPK Ser^399^, Ser^413^, Ser^555^, Ser^588^, Ser^626^	Promoting interaction between cofactors and FOXO3
JNK Thr^447^, Thr^451^	Phosphorylation of FOXO4, translocation of FOXO into the cell nucleus
P38 Ser^294^, Ser^425^, Ser^7^	Phosphorylation of FOXO3 in response to oxidative stress, translocation to the cell nucleus
ERK Ser294, ser344, ser425	Phosphorylation of FOXO3, retention in the cytoplasm

## Data Availability

Not applicable.

## References

[1] Greer E.L., Brunet A. (2005). FOXO transcription factors at the interface between longevity and tumor suppression. Oncogene.

[2] Van Der Vos K.E., Coffer P.J. (2008). FOXO-binding partners: It takes two to tango. Oncogene.

[3] Tzivion G., Dobson M., Ramakrishnan G. (2011). FoxO transcription factors; Regulation by AKT and 14-3-3 proteins. Biochim. Biophys. Acta.

[4] Calissi G., Lam E.W.-F., Link W. (2021). Therapeutic strategies targeting FOXO transcription factors. Nat. Rev. Drug Discov..

[5] Wang Z., Yu T., Huang P. (2016). Post-translational modifications of FOXO family proteins (Review). Mol. Med. Rep..

[6] Yao S., Mahmud Z., Sachini N., Aimjongjun S., Saavedra-García P., Lam E.W.-F. (2019). Characterization of FOXO Acetylation. Methods Mol. Biol..

[7] Yang H., Yan B., Liao D., Huang S., Qiu Y. (2015). Acetylation of HDAC1 and degradation of SIRT1 form a positive feedback loop to regulate p53 acetylation during heat-shock stress. Cell Death Dis..

[8] Grumati P., Dikic I. (2018). Ubiquitin signaling and autophagy. J. Biol. Chem..

[9] Brenkman A.B., de Keizer P., Broek N.J.F.V.D., Jochemsen A.G., Burgering T.B.M. (2008). Mdm2 Induces Mono-Ubiquitination of FOXO4. PLoS ONE.

[10] Yamagata K., Daitoku H., Takahashi Y., Namiki K., Hisatake K., Kako K., Mukai H., Kasuya Y., Fukamizu A. (2008). Arginine Methylation of FOXO Transcription Factors Inhibits Their Phosphorylation by Akt. Mol. Cell.

[11] Butt A.M., Feng D., Idrees M., Tong Y., Lu J. (2012). Computational identification and modeling of crosstalk between phosphorylation, O-β-glycosylation and methylation of FOXO3 and implications for cancer therapeutics. Int. J. Mol. Sci..

[12] Ren H., Shao Y., Wu C., Ma X., Lv C., Wang Q. (2020). Metformin alleviates oxidative stress and enhances autophagy in diabetic kidney disease via AMPK/SIRT1-FoxO1 pathway. Mol. Cell. Endocrinol..

[13] Buteau J., Accili D. (2007). Regulation of pancreatic beta-cell function by the forkhead protein FOXO1. Diabetes Obes. Metab..

[14] Talchai C., Xuan S., Lin H.V., Sussel L., Accili D. (2012). Pancreatic β cell dedifferentiation as a mechanism of diabetic β Cell failure. Cell.

[15] Honzawa N., Fujimoto K. (2021). The Plasticity of Pancreatic β-Cells. Metabolites.

[16] Wang Y., Guan Y., Xue L., Liu J., Yang Z., Nie C., Yan Y., Liu S., Sun J., Fan M. (2021). l-Arabinose suppresses gluconeogenesis through modulating AMP-activated protein kinase in metabolic disorder mice. Food Funct..

[17] Yan D., Cai Y., Luo J., Liu J., Li X., Ying F., Xie X., Xu A., Ma X., Xia Z. (2020). FOXO1 contributes to diabetic cardiomyopathy via inducing imbalanced oxidative metabolism in type 1 diabetes. J. Cell. Mol. Med..

[18] Peserico A., Chiacchiera F., Grossi V., Matrone A., Latorre D., Simonatto M., Fusella A., Ryall J.G., Finley L.W.S., Haigis M.C. (2013). A novel AMPK-dependent FoxO3A-SIRT3 intramitochondrial complex sensing glucose levels. Cell Mol. Life Sci..

[19] Lee S., Dong H.H. (2017). FOXO integration of insulin signaling with glucose and lipid metabolism. J. Endocrinol..

[20] Singh V., Ubaid S. (2020). Role of Silent Information Regulator 1 (SIRT1) in Regulating Oxidative Stress and Inflammation. Inflammation.

[21] Kibbe C., Chen J., Xu G., Jing G., Shalev A. (2013). FOXO1 Competes with carbohydrate response element-binding protein (ChREBP) and inhibits thioredoxin-interacting protein (TXNIP) transcription in pancreatic beta cells. J. Biol. Chem..

[22] Kitamura Y.I., Kitamura T., Kruse J.P., Raum J.C., Stein R., Gu W., Accili D. (2005). FOXO1 protects against pancreatic beta cell failure through NeuroD and MafA induction. Cell Metab..

[23] Furuyama T., Kitayama K., Yamashita H., Mori N. (2003). Forkhead transcription factor FOXO1 (FKHR)-dependent induction of PDK4 gene expression in skeletal muscle during energy deprivation. Biochem. J..

[24] Gupta A., Stocker H. (2020). FoxO suppresses endoplasmic reticulum stress to inhibit growth of Tsc1-deficient tissues under nutrient restriction. eLife.

[25] Bowker-Kinley M.M., Davis W.I., Wu P., Harris R.A., Popov K.M. (1998). Evidence for existence of tissue-specific regulation of the mammalian pyruvate dehydrogenase complex. Biochem. J..

[26] Gopal K., Saleme B., Al Batran R., Aburasayn H., Eshreif A., Ho K.L., Ma W.K., Almutairi M., Eaton F., Gandhi M. (2017). FoxO1 regulates myocardial glucose oxidation rates via transcriptional control of pyruvate dehydrogenase kinase 4 expression. Am. J. Physiol. Heart Circ. Physiol..

[27] Lee S.X., Heine M., Schlein C., Ramakrishnan R., Liu J., Belnavis G., Haimi I., Fischer A.W., Ginsberg H.N., Heeren J. (2018). FoxO transcription factors are required for hepatic HDL cholesterol clearance. J. Clin. Investig..

[28] Tsuchida A., Yamauchi T., Ito Y., Hada Y., Maki T., Takekawa S., Kamon J., Kobayashi M., Suzuki R., Hara K. (2004). Insulin/Foxo1 pathway regulates expression levels of adiponectin receptors and adiponectin sensitivity. J. Biol. Chem..

[29] Mezza T., Shirakawa J., Martinez R., Hu J., Giaccari A., Kulkarni R.N. (2016). Nuclear Export of FoxO1 Is Associated with ERK Signaling in β-Cells Lacking Insulin Receptors. J. Biol. Chem..

[30] Chen Y.-J., Chen C.-C., Li T.-K., Wang P.-H., Liu L.-R., Chang F.-Y., Wang Y.-C., Yu Y.-H., Lin S.-P., Mersmann H.J. (2012). Docosahexaenoic acid suppresses the expression of FoxO and its target genes. J. Nutr. Biochem..

[31] Miao C., Li Y., Zhang X. (2019). The functions of FoxO transcription factors in epithelial wound healing. Australas. J. Dermatol..

[32] Saoncella S., Tassone B., Deklic E., Avolio F., Jon C., Tornillo G., Luca E., Iorio E., Piva R., Cabodi S. (2014). Nuclear Akt2 opposes limbal keratinocyte stem cell self-renewal by repressing a FOXO-mTORC1 signaling pathway. Stem Cells..

[33] Zhang C., Lim J., Jeon H.H., Xu F., Tian C., Miao F., Hameedaldeen A., Graves D.T. (2017). FOXO1 deletion in keratinocytes improves diabetic wound healing through MMP9 regulation. Sci. Rep..

[34] Ponugoti B., Xu F., Zhang C., Tian C., Pacios S., Graves D.T. (2013). FOXO1 promotes wound healing through the up-regulation of TGF-β1 and prevention of oxidative stress. J. Cell Biol..

[35] González-Molero I., Rojo-Martínez G., Morcillo S., Gutiérrez-Repiso C., Rubio-Martín E., Almaraz M.C., Olveira G., Soriguer F. (2012). Vitamin D and incidence of diabetes: A prospective cohort study. Clin. Nutr..

[36] Pittas A.G., Nathan D.M., Nelson J., Hu F., Mitri J., Dawson-Hughes B., Diabetes Prevention Program Research Group (2012). Plasma 25-hydroxyvitamin D and progression to diabetes in patients at risk for diabetes: An ancillary analysis in the diabetes prevention program. Diabetes Care..

[37] Forouhi N.G., Ye Z., Rickard A.P., Khaw K.T., Luben R., Langenberg C., Wareham N.J. (2012). Circulating 25-hydroxyvitamin D concentration and the risk of type 2 diabetes: Results from the European Prospective Investigation into Cancer (EPIC)-Norfolk cohort and updated meta-analysis of prospective studies. Diabetologia.

[38] Borissova A.M., Tankova T., Kirilov G., Dakovska L., Kovacheva R. (2003). The effect of vitamin D3 on insulin secretion and peripheral insulin sensitivity in type 2 diabetic patients. Int. J. Clin. Pract..

[39] Naharci I., Bozoglu E., Kocak N., Doganci S., Doruk H., Serdar M. (2012). Effect of vitamin D on insulin sensitivity in elderly patients with impaired fasting glucose. Geriatr. Gerontol. Int..

[40] Nazarian S., Peter J.V.S., Boston R.C., Jones S.A., Mariash C.N. (2011). Vitamin D3 supplementation improves insulin sensitivity in subjects with impaired fasting glucose. Transl. Res..

[41] Jehle S., Lardi A., Felix B., Hulter H.N., Stettler C., Krapf R. (2014). Effect of large doses of parenteral vitamin D on glycaemic control and calcium/phosphate metabolism in patients with stable type 2 diabetes mellitus: A randomised, placebo-controlled, prospective pilot study. Swiss. Med. Wkly..

[42] Chen S., Villalta S.A., Agrawal D.K. (2015). FOXO1 Mediates Vitamin D deficiency-induced insulin resistance in skeletal muscle. J. Bone Miner. Res..

[43] Xiong Y., Zhang Y., Xin N., Yuan Y., Zhang Q., Gong P., Wu Y. (2016). 1α,25-Dihydroxyvitamin D3 promotes bone formation by promoting nuclear exclusion of the FoxO1 transcription factor in diabetic mice. J. Biol. Chem..

[44] Guo X., Lin H., Liu J., Wang D., Li D., Jiang C., Tang Y., Wang J., Zhang T., Li Y. (2020). 1,25-Dihydroxyvitamin D attenuates diabetic cardiac autophagy and damage by vitamin D receptor-mediated suppression of FoxO1 translocation. J. Nutr. Biochem..

[45] Watała C., Kaźmierczak P., Dobaczewski M., Przygodzki T., Bartuś M., Łomnicka M., Slominska E.M., Duračkova Z., Chlopicki S. (2009). Anti-diabetic effects of 1-methylnicotinamide (MNA) in streptozocin-induced diabetes in rats. Pharmacol. Rep..

[46] Hong S., Moreno-Navarrete J.M., Wei X., Kikukawa Y., Tzameli I., Prasad D., Lee Y., Asara J.M., Fernández-Real J.M., Maratos-Flier E. (2015). Nicotinamide N-methyltransferase regulates hepatic nutrient metabolism through Sirt1 protein stabilization. Nat. Med..

[47] Zhang J., Chen Y., Liu C., Li L., Li P. (2020). N^1^-Methylnicotinamide Improves Hepatic Insulin Sensitivity via Activation of SIRT1 and Inhibition of FOXO1 Acetylation. J. Diabetes Res..

[48] Maeda H., Hosokawa M., Sashima T., Murakami-Funayama K., Miyashita K. (2009). Anti-obesity and anti-diabetic effects of fucoxanthin on diet-induced obesity conditions in a murine model. Mol. Med. Rep..

[49] Yang G., Jin L., Zheng D., Tang X., Yang J., Fan L., Xie X. (2019). Fucoxanthin alleviates oxidative stress through Akt/SIRT1/FOXO3α signaling to inhibit Hg-induced renal fibrosis in GMCs. Mar. Drugs.

[50] Li A., Peng R., Sun Y., Liu H., Peng H., Zhang Z. (2018). LincRNA 1700020I14Rik alleviates cell proliferation and fibrosis in diabetic nephropathy via MIR-34a-5p/Sirt1/HIF-1α signaling. Cell Death Dis..

[51] Wang Y.-Q., Cao Q., Wang F., Huang L.-Y., Sang T.-T., Liu F., Chen S.-Y. (2015). SIRT1 Protects Against Oxidative Stress-Induced Endothelial Progenitor Cells Apoptosis by Inhibiting FOXO3a via FOXO3a Ubiquitination and Degradation. J. Cell. Physiol..

[52] Kim-Muller J.Y., Zhao S., Srivastava S., Mugabo Y., Noh H.L., Kim Y.R., Madiraju M., Ferrante A.W., Skolnik E.Y., Prentki M. (2014). Metabolic inflexibility impairs insulin secretion and results in MODY-like diabetes in triple FOXO-deficient mice. Cell Metab..

[53] Filios S.R., Shalev A. (2015). β-cell microRNAs: Small but powerful. Diabetes..

[54] Osmai M., Osmai Y., Bang-Berthelsen C.H., Pallesen E.M.H., Vestergaard A.L., Novotny G.W., Pociot F., Mandrup-Poulsen T. (2016). MicroRNAs as regulators of beta-cell function and dysfunction. Diabetes/Metab. Res. Rev..

[55] Li Y., Deng S., Peng J., Wang X., Essandoh K., Mu X., Peng T., Meng Z.-X., Fan G.-C. (2019). MicroRNA-223 is essential for maintaining functional β-cell mass during diabetes through inhibiting both FOXO1 and SOX6 pathways. J. Biol. Chem..

[56] Peng S., Xiao W., Ju D., Sun B., Hou N., Liu Q., Wang Y., Zhao H., Gao C., Zhang S. (2019). Identification of entacapone as a chemical inhibitor of FTO mediating metabolic regulation through FOXO1. Sci. Transl. Med..

[57] Akao M., Ohler A., O’Rourke B., Marbán E. (2001). Mitochondrial ATP-Sensitive potassium channels inhibit apoptosis induced by oxidative stress in cardiac cells. Circ. Res..

[58] Qi Y., Xu Z., Zhu Q., Thomas C., Kumar R., Feng H., Dostal D.E., White M.F., Baker K.M., Guo S. (2013). Myocardial Loss of IRS1 and IRS2 Causes Heart Failure and Is Controlled by p38α MAPK During Insulin Resistance. Diabetes.

[59] Duan P., Wang J., Li Y., Wei S., Su F., Zhang S., Duan Y., Wang L., Zhu Q. (2018). Opening of mitoKATP improves cardiac function and inhibits apoptosis via the AKT-Foxo1 signaling pathway in diabetic cardiomyopathy. Int. J. Mol. Med..

[60] Nagashima T., Shigematsu N., Maruki R., Urano Y., Tanaka H., Shimaya A., Shimokawa T., Shibasaki M. (2010). Discovery of Novel Forkhead Box O1 inhibitors for treating Type 2 diabetes: Improvement of fasting glycemia in diabetic *db/db* mice. Mol. Pharmacol..

[61] Shi Y., Fan S., Wang D., Huyan T., Chen J., Chen J., Su J., Li X., Wang Z., Xie S. (2018). FOXO1 inhibition potentiates endothelial angiogenic functions in diabetes via suppression of ROCK1/Drp1-mediated mitochondrial fission. Biochim. Biophys. Acta. Mol. Basis. Dis..

[62] Yu F., Wei R., Yang J., Liu J., Yang K., Wang H., Mu Y., Hong T. (2018). FoxO1 inhibition promotes differentiation of human embryonic stem cells into insulin producing cells. Exp. Cell Res..

[63] Park J., Hwang I., Kim S.-J., Youn S.-W., Hur J., Kim H.-S. (2018). Atorvastatin prevents endothelial dysfunction in high glucose condition through Skp2-mediated degradation of FOXO1 and ICAM-1. Biochem. Biophys. Res. Commun..

[64] Lee H.-Y., Youn S.-W., Cho H.-J., Kwon Y.-W., Lee S.-W., Kim S.-J., Park Y.-B., Oh B.-H., Kim H.-S. (2013). FOXO1 impairs whereas statin protects endothelial function in diabetes through reciprocal regulation of Krüppel-like factor 2. Cardiovasc. Res..

[65] Atkins G.B., Jain M.K. (2007). Role of Krüppel-like transcription factors in endothelial biology. Circ. Res..

[66] Jang I.-A., Kim E.N., Lim J.H., Kim M.Y., Ban T.H., Yoon H.E., Park C.W., Chang Y.S., Choi B.S. (2018). Effects of resveratrol on the renin-angiotensin system in the aging kidney. Nutrients.

[67] Galiniak S., Aebisher D., Bartusik-Aebisher D. (2019). Health benefits of resveratrol administration. Acta Biochim. Pol..

[68] Wang X., Meng L., Zhao L., Wang Z., Liu H., Liu G., Guan G. (2017). Resveratrol ameliorates hyperglycemia-induced renal tubular oxidative stress damage via modulating the SIRT1/FOXO3a pathway. Diabetes Res. Clin. Pract..

[69] Li P., Song X., Zhang D., Guo N., Wu C., Chen K., Liu Y., Yuan L., Chen X., Huang X. (2020). Resveratrol improves left ventricular remodeling in chronic kidney disease via Sirt1-mediated regulation of FoxO1 activity and MnSOD expression. BioFactors.

[70] Chen P., Shi X., Xu X., Lin Y., Shao Z., Wu R., Huang L. (2018). Liraglutide ameliorates early renal injury by the activation of renal FoxO1 in a type 2 diabetic kidney disease rat model. Diabetes Res. Clin. Pract.

[71] Rena G., Hardie D.G., Pearson E.R. (2017). The mechanisms of action of metformin. Diabetologia.

[72] Al-Masri M., Krishnamurthy M., Li J., Fellows G.F., Dong H.H., Goodyer C.G., Wang R. (2010). Effect of forkhead box O1 (FOXO1) on beta cell development in the human fetal pancreas. Diabetologia.

[73] Wu L., Li H., Jia C.Y., Cheng W., Yu M., Peng M., Zhu Y., Zhao Q., Dong Y.W., Shao K. (2012). MicroRNA-223 regulates FOXO1 expression and cell proliferation. FEBS Lett..

[74] Kousteni S., Kousteni S. (2012). The PDK1-FOXO1 signaling in adipocytes controls systemic insulin sensitivity through the 5-lipoxygenase-leukotriene B4 axis. FOXO1, the transcriptional chief of staff of energy metabolism. Bone.

